# Beyond the conventional risk factors in the management of atherosclerosis in women: a call to action for careful consideration of obesity and inflammation

**DOI:** 10.1093/ehjopen/oeag055

**Published:** 2026-04-27

**Authors:** Mirvat Alasnag, Antonia Sambola, Izabella Uchmanowicz, Marta Kaluzna, Pierre François Sabouret, Laura Serrano, Valeria Paradies, Biljana Parapid, Shrilla Banerjee, Anne Bachelot, Martine Gilard, Michał Hawranek, Vijay Kunadian, Alaide Chieffo, Roxana Mehran, Stephane Manzo Silberman

**Affiliations:** Cardiac Center, King Fahd Armed Forces Hospital, PO Box 126418, Alcorniche Jeddah 21372, Saudi Arabia; d'Hebron Hospital Universitari Vall d'Hebron Paseo Vall d'Hebron, 119-129 08035 Barcelona, Spain; Department of Laboratory Diagnostics, 4th Military Hospital of Wroclaw, Weigla 5, 50-981, Wroclaw, Poland; 1st Department of Cardiology, Medical University in Poznan, 10 Fredry Street, Poznan, Poland; Institute of Cardiology Hôpital Pitié-Salpêtrière (AP-HP), ACTION Study Group, Sorbonne University, 47–83 Boulevard de l'Hôpital, 75013 Paris, France; Cardiocean Clinic, Puilboreau, 13080 Aix-en-Provence, France; Cardiology Department, La Rochelle Saint Louis Hospital, Rue Du Docteur Schweitzer 17019, La Rochelle Cedex 1, France; Cardiology Department, Maasstad Hospital, Maasstad Ziekenhuis Maasstadweg 21, 3079 DZ Rotterdam, KvK 24299846, Rotterdam, The Netherlands; Division of Cardiology, University Clinical Center of Serbia, Faculty of Medicine, University of Belgrade, Studentski trg 1, 11102 Belgrade 3, Serbia; Department of Cardiology, Surrey and Sussex Healthcare, East Surrey Hospital, Canada Avenue, Redhill, Surrey RH1 5RH, UK; AP-HP, Hôpitaux Universitaires Pitié-Salpêtrière, Service d’Endocrinologie et Médecine de la Reproduction, Sorbonne Université, 47-83 boulevard de l’Hôpital, Paris 75013, France; Cardiology Department, CHU Brest, Boulevard Tanguy Prigent, Brest 29209, France; 3rd Department of Cardiology, Faculty of Medical Sciences in Zabrze, Medical University of Silesia, 5 Poniatowskiego Street, 40-055 Katowice, Poland; Translational and Clinical Research Institute, Faculty of Medical Sciences, Newcastle University and Cardiothoracic Centre, Freeman Hospital, Newcastle upon Tyne Hospitals, NHS Foundation Trust, Royal Victoria Infirmary, Queen Victoria Road, Newcastle upon Tyne NE1 4LP, UK; Ospedale San Raffaele, Università Vita-Salute San Raffaele Facoltà di Medicina e Chirurgia, Università degli Studi di Napoli Federico II Dipartimento di Medicina Clinica e Chirurgia, Via Olgettina, 60, 20132 Milano MI, Italy; Mount Sinai Fuster Heart Hospital, Icahn School of Medicine at Mount Sinai, 190 Fifth Avenue and 1468 Madison Avenue, New York, USA; Institute of Cardiology Hôpital Pitié-Salpêtrière (AP-HP), ACTION Study Group, Sorbonne University, 47–83 Boulevard de l'Hôpital, 75013 Paris, France

**Keywords:** Obesity, Inflammation, Atherosclerosis, Women, Risk factors

## Abstract

Conventional risk factors do not completely explain the burden of atherosclerotic cardiovascular disease (ASCVD) in women. Obesity and chronic low-grade inflammation are recognized as important contributors to atherosclerotic risk. This viewpoint explains the role of obesity-driven inflammation in the development and management of atherosclerosis in women. Abdominal obesity promotes a proinflammatory and prothrombotic environment that accelerates atherogenesis. Women with higher inflammatory biomarker levels and experience sex-specific risk-modifying stages, such as polycystic ovary syndrome, pregnancy-related conditions, and menopause, have higher risk for cardiovascular events, including atherosclerosis. We review evidence for antiobesity and anti-inflammatory therapies. Integrating obesity and inflammation into ASCVD care in women requires targeted screening, risk assessment, and sex-stratified research approaches. This viewpoint highlights the relation between obesity and atherosclerosis.

## Introduction and basic concepts

Conventional cardiovascular (CV) risk factors, such as hypertension, dyslipidaemia, diabetes mellitus, smoking, and sedentary lifestyle, form the hallmark of atherosclerotic cardiovascular disease (ASCVD). However, these factors alone do not account for the CV risk observed in women, highlighting the contribution of other risk factors, including obesity and chronic systemic inflammation.^1,2^The Global Burden of Disease (GBD) Obesity Collaborators estimated that the rate of obesity has doubled over the past two decades, with ∼39–49% of the world’s population (2.8–3.5 billion people) classified as overweight or obese.^3^

Obesity is a multifactorial condition, with its pathogenesis consisting of environmental, socioeconomic, and biological determinants. The conventional definition of obesity relies on body mass index (BMI). While BMI is useful at a population level, it fails to capture sex-specific differences in fat distribution and cardiometabolic risk. Abdominal (central) obesity is assessed by waist circumference (>88 cm in women). It is more accurate to reflect visceral adiposity and is more strongly associated with insulin resistance, endothelial dysfunction, and a proatherogenic inflammatory state.^4^ Obesity is a proinflammatory state, characterized by increased levels of inflammatory cytokines, such as interleukin-6 (IL-6) and tumour necrosis factor-alpha (TNF-α).^5^ This inflammatory state exacerbates traditional risk factors, leading to increased risk of CV events.^5^ This viewpoint highlights the role of obesity and chronic inflammation in the development of atherosclerosis. By understanding this relationship, targeted interventions can reduce CV events, including atherosclerosis.

**Figure 1:**
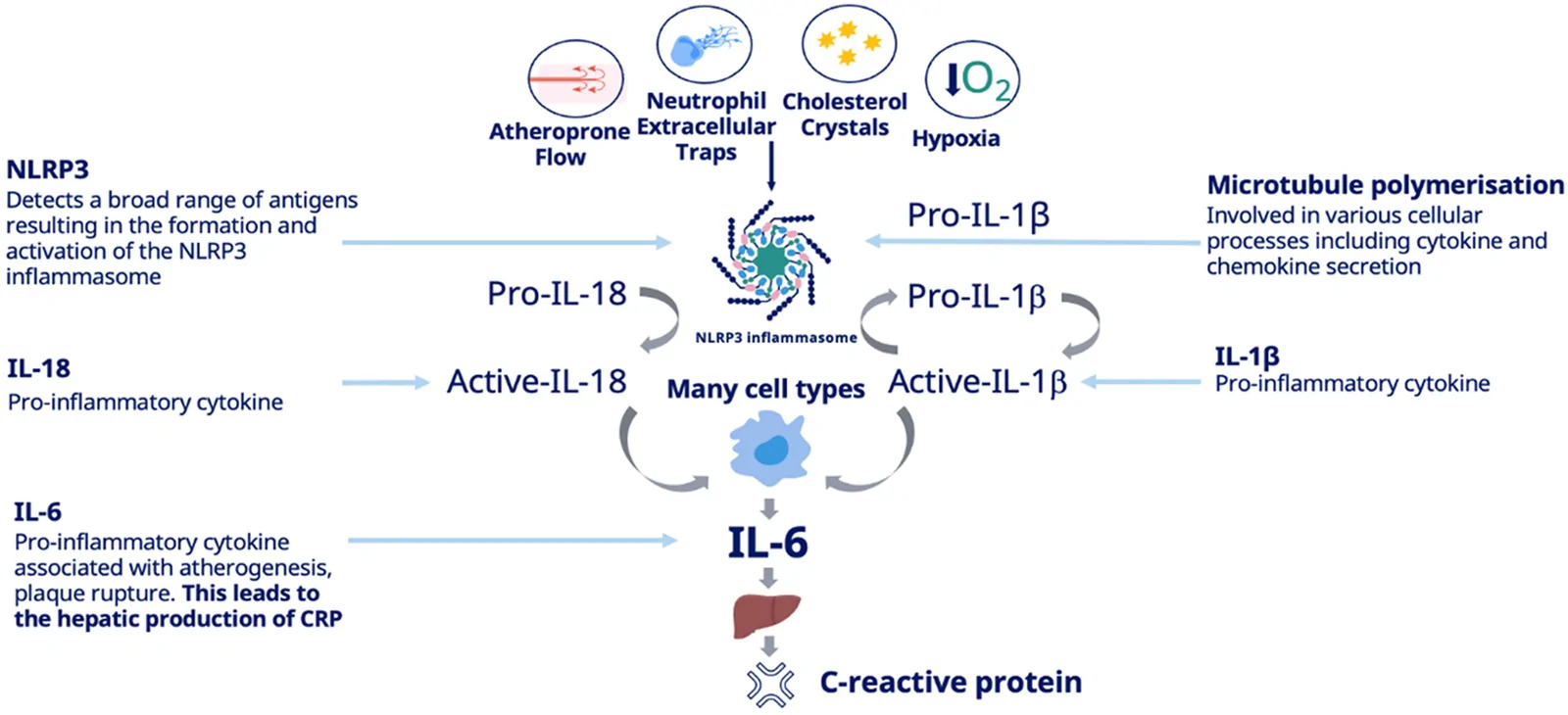
Role of the NLRP3 inflammasome in atherosclerosis. IL, interleukin; NLRP3, NOD (nucleotide oligomerization domain)-, LRR (leucine-rich repeat)-, and PYD (pyrin domain)-containing protein 3; MMP, matrix metalloproteinase; pro-IL, interleukin precursor; VSMC, vascular smooth muscle cells. Ridker PM et al. Circulation 2020;141:787‒789.

Chronic low-grade systemic inflammation is a key factor in the pathogenesis of ASCVD. Triggers of chronic inflammation include viral infections, rheumatic or autoimmune disorders, cancers, and vascular injury. Vascular injury is interesting as it includes triggers, such as smoking and lipoprotein(A).^6^ The progression and instability of atheroma precipitate acute CV events.^7^ In patients with atherosclerosis, cholesterol and oxidized LDL are the main activators of the NLRP3 (NOD-, LRR-, and pyrin domain-containing protein 3) inflammasome (*Figure 1*). These stimuli promote inflammation, oxidative stress, and cell death within the arterial wall. In addition, these crystals cause atheroprone flow (low shear stress, oscillatory flow, and flow separation), which leads to endothelial dysfunction, inflammation, and accumulation of lipids and immune cells in the arterial wall.^8^

### Interplay between obesity, inflammation, and female-specific conditions across the life course

**Figure 2:**
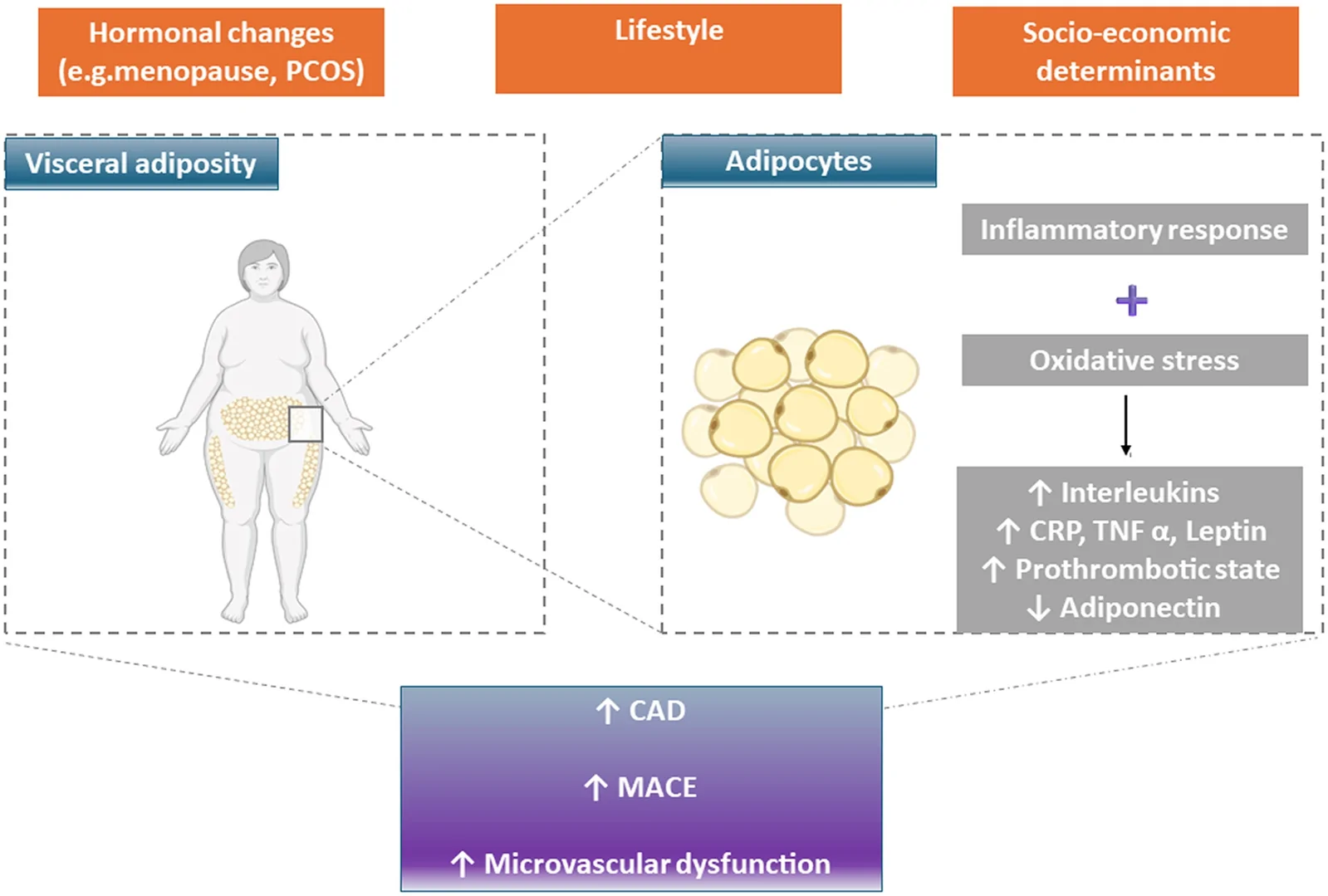
Impact of obesity.

Obesity represents a complex interplay of factors that result in excess weight and fat accumulation (*Figure 2*). These factors include neurobiological, genetic, behavioural, socioeconomic, and psychological factors.^9^ Excess adipose tissue, especially found with visceral fat deposition, secretes cytokines, such as leptin and adiponectin, and dysregulates the secretion of inflammatory adipocytokines, promoting inflammatory cytokines and the proliferation of proinflammatory macrophages.^10,11^ This complex proinflammatory state contributes to increased oxidative stress and the activation of sympathetic pathways, leading to metabolic disturbance and endothelial dysfunction.^12^ Dysregulated adipocytokines can induce insulin resistance, endothelial dysfunction, a prothrombotic state, neurohormonal imbalance, and systemic or local inflammation.^13,14^ Activation of the inflammation pathway, including TNFα, IL-1, IL-6, and plasminogen activator inhibitor-1, raises insulin resistance, modifies lipid metabolism, increases thrombogenicity, decreases the vasoreactivity of the arterial wall, and increases blood pressure (BP).

In women, the biological consequences are more significant.^15^ Premenopausal, nonobese women have lower CV risk compared to men, but obesity significantly worsens a woman’s CV risk profile. The role of inflammation can be detected through higher levels of mean C-reactive protein, which are found in obese women compared to obese men.^16^ Leptin is another adipokine elevated in obesity, which has a different impact according to sex.^16^ Moreover, the protective effect of sexual steroids appears decreased in obesity with a decrease in the oestrogen signalling pathway in endothelial cells, leading to reduced nitric oxide (NO) availability, increased arterial tone, and stiffness.^14^ Androgen excess associated with increased adiposity contributes to an unfavourable CV profile. This is also observed in polycystic ovarian syndrome and the postmenopausal state.^17^ Increased androgen levels (hyperandrogenism) are associated with obesity, hypertension, insulin resistance, and endothelial dysfunction.^17–19^

### Polycystic ovarian syndrome

Hyperandrogenism is considered the primary cause of polycystic ovarian syndrome (PCOS). Between 30 and 50% of patients with PCOS present with metabolic syndrome from adolescence.^20^ Women with PCOS frequently have an atherogenic lipid profile, characterized by elevated triglycerides (TG), very LDL cholesterol (VLDL-C), LDL cholesterol (LDL-C), and reduced HDL cholesterol (HDL-C) levels. These lipid abnormalities are relatively linked to insulin resistance and reflect metabolic and inflammatory pathways that promote atherosclerosis in women.^21^ In a meta-analysis of 30 studies, it was found that women with PCOS have higher LDL-C, non-HDL-C, and TG levels, along with lower HDL-C, compared with age- and weight-matched women without PCOS, even after adjustment for BMI.^22^ This highlights that atherogenic dyslipidaemia in women may arise independently of generalized obesity and the importance of sex-specific metabolic risk modifiers.^22^ The dyslipidaemia observed in PCOS is thought to be from obesity, hyperandrogenism, and insulin resistance. Mechanisms that explain dyslipidaemia in PCOS include impaired insulin signalling, increased hepatic production of apolipoprotein B-containing very LDLs, abnormal lipoprotein lipase activity, and androgen receptor–mediated HDL catabolism. Consistent with this, treatment with the androgen receptor antagonist flutamide has been shown to improve lipid profiles in women with PCOS.^23^

### Endometriosis

The most widely accepted theory for pathophysiology of endometriosis is retrograde menstruation, where menstrual blood containing endometrial cells flows through the fallopian tubes into the pelvic cavity. This allows these cells to implant, proliferate, and cyclically bleed.^24–26^ Other hypotheses, including contributions from endometrial or bone marrow stem cells, lymphovascular spread, and coelomic metaplasia, have been suggested to explain ectopic lesion locations.

Endometriosis and atherosclerosis have common cellular and molecular features. Both involve chronic inflammation, oxidative stress, endothelial dysfunction, and cellular proliferation.^24–26^ Endometriosis is a multifactorial disease affected by hormonal, proinflammatory, proangiogenic, immunologic, and genetic factors.^26^ The proliferation of endometrial fragments relies on systemic oestradiol and altered hormonal signalling pathways.^26,27^ Recruitment of macrophages and activation of cytokines and angiogenic factors promote neovascularization and ectopic lesion growth within a proinflammatory environment.^26,28^ Endometriosis has also been associated with increased arterial stiffness and impaired flow-mediated dilation, a marker of endothelial dysfunction that may improve after surgical intervention.^26,29–31^

### Menopause

Menopause is characterized by an accelerated decline in oestrogen levels, which begins at the age of 35.^32^ The role of oestrogen in the CV system is well established. It contributes to vascular reactivity, BP regulation, endothelial function, cardiac remodelling, and immune modulation. The atheroprotective effects of oestradiol have been studied.^33–37^ The accelerated and sharp decrease in oestrogen levels during menopause causes a variety of systemic effects. It reduces basal metabolic rate and leads to a redistribution of abdominal and subcutaneous fat, with a significant increase in visceral adiposity. The menopausal transition is associated with changes in insulin and glucose metabolism, including insulin resistance and even a decrease in insulin secretion.^38,39^ Moreover, decreased levels of sex hormone-binding globulin (SHBG) and the related increase in circulating testosterone are also associated with insulin resistance. The increase in visceral fat triggers inflammation and contributes to insulin resistance.^40^ Additionally, lipid metabolism changes significantly during the menopausal transition, with increases in TG, total cholesterol, and LDL cholesterol, accompanied by a decrease in HDL cholesterol.^41^ It is also noted that the increase in LDL is independent of age, BMI, and environmental factors.^42^

### Pregnancy

Pregnancy can exacerbate underlying CV risks, especially in women with obesity or systemic inflammation. This leads to complications, such as gestational hypertension and preeclampsia, which are linked to long-term ASCVD risk.^43^ According to a study addressing the impact of obesity on outcomes of pregnancy in women with heart disease, with a sample size of 790 pregnancies, they found that 19% occurred in women with obesity (BMI of 30 kg/m^2^ or higher) and 25% in overweight women (BMI of 25–29.9 kg/m^2^). Women with obesity had a higher incidence of CV events compared with normal-weight women (23% vs. 14%; *P* = 0.006). In a multivariable analysis, both obesity (odds ratio [OR] 1.7; 95% confidence interval [CI] 1.0–2.7) and higher Cardiac Disease in Pregnancy Study II scores (OR 1.7; 95% CI 1.5–1.9) independently predicted CV events. Preeclampsia was more common in women with obesity than in those with normal weight (8% vs. 2%; *P* = 0.001).^43^ It is crucial for women with cardiometabolic risk factors to undergo preconception CV assessment, including BP, lipid profile, BMI, insulin resistance, and inflammatory biomarkers. Early identification and management of these modifiable risks can improve maternal and foetal outcomes and reduce pregnancy complications and ASCVD risk.

### Management

Treatment of obesity has the potential to improve CV risk factors and reduce CV events. Current recommendations emphasize regular assessment of BMI, CV risk factors, established cardiovascular disease (CVD), and readiness for weight loss. For individuals with a BMI of ≥30 kg/m^2^, or ≥27 kg/m^2^ in the presence of CVD or cardiometabolic risk factors, comprehensive lifestyle interventions represent the first-line therapeutic approach.^44,45^ Bariatric surgery may be considered for individuals with a BMI of ≥40 kg/m^2^, or ≥35 kg/m^2^ with CVD. Pharmacological therapies may be added to patients with an inadequate response to lifestyle interventions or in those with established CVD. Lifestyle and behavioural strategies, including nutritional counselling and structured physical activity, form the foundation of all obesity treatment approaches and are also recommended for the primary prevention of CVD. However, evidence suggests that clinically meaningful CV benefit generally requires weight loss exceeding 10% of baseline body weight.^46^ Lifestyle interventions alone often fail to achieve or sustain this degree of weight loss and are frequently complicated by weight regain.^47,48^

### Nonpharmacological interventions

Obesity management involves lifestyle changes (physical health, nutritional health, emotional health), even if therapeutic or surgical treatment is considered. Obesity is a considerable burden, and the physical and mental quality of life for overweight women or women living with obesity is impaired. One way to improve their quality of life and their CV and global prognosis is to practice regular physical activity. It is recommended to practice at least 150–300 min per week of moderate endurance physical activity, spread over three to five sessions per week, or 75–150 min per week of vigorous physical activity (PA), or a combination of both intensities. It is also recommended to practice resistance PA two to three times per week.^49,50^

In the middle-aged female adult population, including those suffering from obesity, there are barriers related to daily life to PA, such as the combination of family and professional life, as well as caring for children, family, and household chores, which alter the desire and motivation to engage in PA. Women also feel less justified in engaging in endurance PA. One solution is to offer a variety of physical activities, supervised by a PA instructor, combining endurance and resistance, for example, walking, running, cycling, playing tennis or team sports, gymnastics classes, and resistance training.^51^ A weekly session of vigorous PA or high-intensity interval training (HIIT) is associated with a lower risk of hypertension and obesity and improves cardiorespiratory fitness (VO_2_ peak), cardiac stroke volume, BP, body composition, and lipid profile and is likely to provide similar benefits to Depot MedroxyProgesterone Acetate in terms of body fat reduction in women.^52^

Regarding diet, calorie reduction along with a Mediterranean-style diet is recommended. The goal is to meet the needs and preferences of women.^49,50^ A low-calorie diet, without a prescription for PA, is sufficient for short-term weight loss, but PA is essential for changing body composition in women in situation of obesity. Fat mass is lower in women who engage in moderate exercise than in those following a low-calorie diet alone. Tailored exercise program is more effective at reducing body fat and maintaining muscle mass.^53^ Mindful eating is associated with weight loss but is not superior to other dietary control techniques for women.^54^ Mental and emotional health should also be addressed; outpatient counselling, cognitive–behavioural therapy, support groups, and psychosocial support should be encouraged for better weight loss, to reduce binge eating in women, who are more often affected by this phenomenon than men, and also to limit anxiety and depression associated with obesity or overweight.^49,55,56^

E-health interventions can increase PA and improve obesity-related outcomes by monitoring weight, food intake, and emotions. All these interventions can be encouraged by participation in long-term programmes, support groups, and psychological support, in addition to staying in CV rehabilitation centres that promote programmes for patients who are classified as overweight or obese, or centres specialized in metabolic management.^49,57,58^

## Pharmacological interventions

### Anti-inflammatory therapies

Obesity exacerbates the release of proinflammatory cytokines, such as leptin, IL-6, and TNF-α. These proinflammatory cytokines promote oxidative stress, induce a prothrombotic state, and reduce levels of anti-inflammatory adiponectin. This creates a chronic low-grade inflammatory state. This sustained inflammatory environment increases the risk of incident coronary artery disease (CAD) and major adverse cardiovascular events (MACE) in women with obesity.^59^

Social determinants, such as discrimination and chronic stress, have also been related to elevated inflammatory markers in women, which may further elevate their CVD risk beyond traditional factors.^60^ Anti-inflammatory therapies, especially those targeting the central IL-6 signalling pathway, may serve as promising treatment strategies to reduce the risk of myocardial infarction (MI). Colchicine is a well-established anti-inflammatory drug. The Canakinumab Anti-inflammatory Thrombosis Outcomes Study (CANTOS) provided the first direct evidence that targeting inflammation reduces atherosclerotic CV events independent of lipid lowering. They found that canakinumab produced a dose-dependent and significant reduction in systemic inflammation, with high-sensitivity C-reactive protein levels decreasing more than placebo by 26, 37, and 41% in the 50, 150, and 300 mg groups, respectively (all *P* < 0.001). Interleukin-6 levels showed similar reductions. In contrast, canakinumab did not lower LDL or HDL cholesterol and was associated with a small (4–5%) increase in TG, indicating that its benefits are independent of lipid modification.^61^

Multiple large-scale clinical trials (e.g. COLCOT and LoDoCo2) have demonstrated that long-term, low-dose colchicine significantly lowers the incidence of MACE in patients with CAD.^62–65^ A small study of colchicine also showed that colchicine resulted in favourable effects on coronary plaque stabilization at optical coherence tomography in patients with acute coronary syndrome.^65^ Analyses from COLCOT/LoDoCo2 indicate that the hazard reduction for adverse CV events appeared statistically significant in men as colchicine trials typically have low female enrolment and are underpowered to detect real sex differences, limiting definitive conclusions on differential efficacy by sex.^64,65^

Ziltivekimab is a novel monoclonal antibody targeting IL-6, a key cytokine in the inflammatory cascade linked to atherosclerosis and CV risk. Early-phase clinical trials have shown that ziltivekimab significantly reduces inflammatory biomarkers (such as C-reactive protein and fibrinogen) in patients with chronic kidney disease and elevated CV risk.^65–67^ Ongoing large-scale trials are investigating its impact on MACE, particularly in high-risk populations. While most clinical trials of anti-inflammatory drugs, such as colchicine and IL-6 inhibitors (e.g. ziltivekimab), have not been exclusively conducted in women, the pathophysiological rationale for their use is strong, given the higher inflammatory burden in obese women with CVD.^65^

Evidence that anti-inflammatory CV therapies differ in efficacy by sex among patients with atherosclerosis is limited. Women remain underrepresented in major trials.^64,65^ Sex-stratified analyses are often underpowered or observational, preventing clear conclusions. Therefore, clinicians should interpret current subgroup findings and encourage future trials designed with sex-specific analyses.

### Clinical and research implications

Evidence guiding the management of atherosclerosis in women remains limited due to underrepresentation of women in CV trials and inadequate stratification by sex-specific factors. Clinically, systematic assessment of central obesity and inflammatory burden is needed, particularly during high-risk life stages, such as pregnancy and menopause. Future research should prioritize sex-disaggregated analyses, with prespecified stratification by menopausal status, hormonal exposure, and inflammatory biomarkers, to support the development of evidence-based, sex-specific strategies for atherosclerosis prevention and management in women.

### Role of hormone replacement therapy

Hormone replacement therapy (HRT) plays a significant role in modulating CV risk in postmenopausal women, particularly those with obesity. The loss of oestrogen after menopause contributes to increased visceral fat accumulation, insulin resistance, and systemic inflammation—all of which accelerate the development of atherosclerosis.^68,69^ Oestrogen has protective vascular properties. It enhances endothelial function, promotes vasodilation through NO synthesis, improves lipid profile by lowering LDL and raising HDL, and suppresses inflammatory signalling.^70,71^ Hormone replacement therapy, especially when initiated early in the menopausal transition, can restore many of these benefits. In obese women, it helps reduce central adiposity, improve glucose metabolism, and lower circulating markers of inflammation.^72^

The KEEPS and ELITE trials showed that early HRT initiation slowed carotid intima–media thickness progression and reduced inflammatory markers.^73,74^ These effects collectively support its potential role in reducing CV risk. The formulation and route of administration matter. Transdermal oestrogen is particularly beneficial for women with higher BMI, as it avoids first-pass hepatic metabolism and is associated with a lower risk of thromboembolic events compared to oral oestrogen.^75^ When appropriately tailored, HRT can be safely integrated into the CV risk management of postmenopausal women, particularly those with early metabolic dysfunction. Timing is also critical. Starting HRT within 10 years of menopause or before age 60 is associated with more favourable CV outcomes than initiating it later.^76^

Though not a universal solution, HRT offers an underused opportunity to address the intertwined roles of hormonal decline, obesity, and inflammation in the development of atherosclerosis.^77^ It is important to note that HRT may carry potential risks, including an increased incidence of thromboembolic events, strokes, and select cancers (e.g. breast and endometrial).^78,79^ Treatment regimens should be individually tailored, with careful monitoring of therapy duration and patient health status to minimize these complications.

### Antiobesity therapies

Individuals who are overweight or obese are at an increased risk of CV morbidity and mortality.^80^ The concepts of ‘obesity paradox’ and ‘metabolically healthy obesity’, which have suggested a more favourable prognosis in certain subgroups, have been increasingly questioned considering recent evidence.^81^ Large epidemiological studies have demonstrated a nonlinear relationship between BMI and total mortality.^82^ For example, the HUNT study and the UK Biobank reported that every 1 unit increase in BMI above 25 kg/m^2^ was associated with a 5 and 9% increase in total mortality, respectively.^82^ This excess risk is primarily driven by CVD death, particularly due to MI and stroke.^81,82^

**Figure 3:**
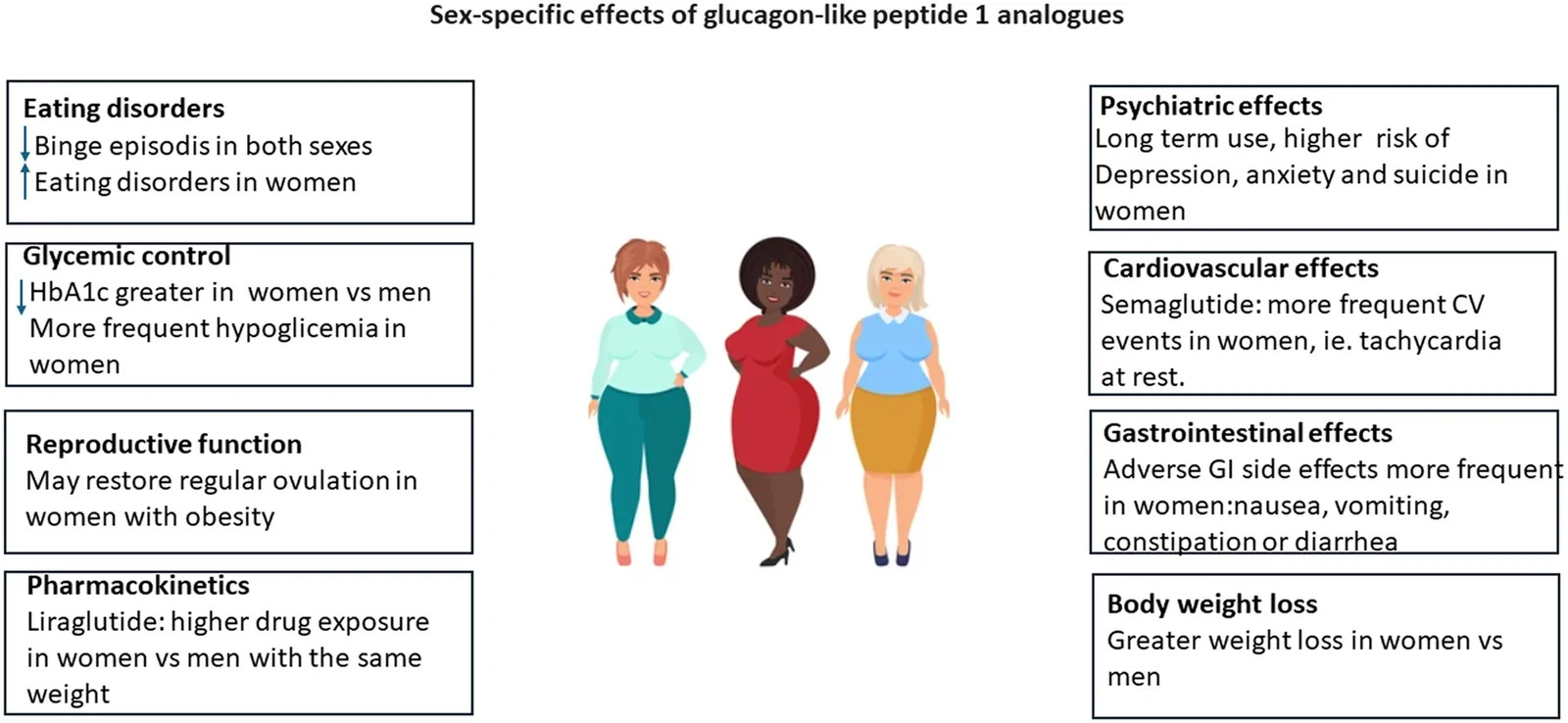
Sex specific effects of glucagon like peptide 1 analogues

Several pharmacological options are currently approved for the long-term management of obesity (*Figure 3*).^83^ Earlier agents, such as phentermine–topiramate, naltrexone–bupropion, and orlistat, achieved only modest weight loss and showed no consistent CV benefits. More recently, newer therapies, including liraglutide (3 mg), semaglutide (2.4 mg), and tirzepatide (5, 10, or 15 mg), have demonstrated a substantially higher weight loss along with evidence of CV benefit.^83^ Selection among these agents should be based on costs and availability as primary determinants while also considering patients’ clinical profile, individual needs, and risk factors (*Table 1*).

**Table 1:** Current indicated pharmacological agents for the management of obesity

Pharmacological agent	Benefit	Undesirable effect	Sex-specific efficacy
Semaglutide 2.4 mg	May be preferred over other approved obesity management medication Has glucoregulatory benefit and is approved for T2DM	Causes nausea and vomiting—gradual dose titration may mitigate it ﻿GLP-1 RAs have been associated with increased risk of pancreatitis and gallbladder diseases	Women treated with semaglutide 2.4 mg lost a mean of −9.6% body weight vs. −7.2% in men^84^
Liraglutide 3 mg	May be preferred over other approved obesity management medication Has glucoregulatory benefit and is approved for T2DM	Causes nausea and vomiting—gradual dose titration may mitigate it ﻿Liraglutide has been associated with increased risk of pancreatitis and gallbladder diseases	Men achieved greater weight loss and BMI reduction than women^85^
Tirzepatide 5, 10, and 15 mg	May be preferred over other approved obesity management medication Has glucoregulatory benefit and is approved for T2DM Neoplasia syndrome type 2 ﻿Monitor for depression or suicidal thoughts	Causes nausea, vomiting, and diarrhoea—potentially severe May cause acute pancreatitis and acute gallbladder disease Use with caution in patients with a history of pancreatitis or severe gastrointestinal diseases, including gastroparesis, due to limited studies Use with caution and monitor patients with a history of diabetic retinopathy ﻿Contraindication in patients with personal or family history of medullary thyroid carcinoma and in patients with multiple endocrine neoplasia	Females have higher odds of achieving very large reductions (≥20%), while males sometimes show faster early per cent loss within responder categories^86^
Phentermine–topiramate	Preferred in patients with comorbid migraines	Avoided in patients with a history of CVD and uncontrolled systemic hypertension Teratogenic—women of childbearing potential should use effective contraception Monitor BP and HR periodically with phentermine	Not reported
Naltrexone–bupropion	﻿Considered in patients who are attempting smoking cessation and in patients with depression﻿	Avoided in patients with seizure disorders and used with caution in patients at risk of seizures Not recommended in patients with severe renal impairment (<30 mL/min) Should not be used concomitantly with opiate medications Contraindicated in patients with uncontrolled systemic hypertension Monitor BP and HR periodically, especially in the first 12 weeks of treatment	Literature does not report sex-stratified outcomes for the combination; the only supplied sex-stratified result is for naltrexone alone, favouring women^87^

Adapted from Grunvald et al., 2022^2^; Garvey et al., 2016^15^; Zepbound PI, 2022^19^; and Mounjaro EPAR, 2023.^20^

BP, blood pressure; CVD, cardiovascular disease; GLP-1 RA, glucagon-like peptide-1 receptor agonists; HR, heart rate; T2DM, Type 2 diabetes mellitus.

These three newer agents, liraglutide, semaglutide, and tirzepatide, have demonstrated sustained and superior weight reduction in randomized clinical trials (RCTs). A recent meta-analysis on liraglutide 3 mg ﻿ reported significant weight loss compared to placebo, with a higher likelihood of achieving at least 5 and 10% weight loss. In the Semaglutide Treatment Effect in People with Obesity (STEP) programme, semaglutide 2.4 mg plus lifestyle intervention achieved greater weight reduction as well as weight-loss targets compared with lifestyle intervention alone. In the STEP 1 trial, nondiabetic individuals receiving semaglutide 2.4 mg plus lifestyle intervention achieved a 14.9% body weight reduction from baseline, vs.2.4% in the lifestyle intervention arm, over 68 weeks.

Tirzepatide, a novel first-in-class dual glucose-dependent insulinotropic polypeptide (GIP) and glucagon-like peptide-1 receptor agonist (GLP-1 RA), has been evaluated at doses of 5, 10, and 15 mg, respectively in the SURMOUNT-1 trial. This trial included patients with obesity or overweight and at least one weight-related complication, excluding diabetes. Participants achieved mean weight reductions of 11.9, 16.4, and 17.8% for 5, 10, and 15 mg doses, respectively, at 72 weeks.^88^

Ongoing RCTs are evaluating the potential CV benefits and the safety of this dual agonist. Several studies have shown that weight loss can reduce MACE. The Look AHEAD trial highlighted the role of lifestyle interventions in improving CV outcomes.^46^ Notably, clinical benefits of semaglutide appeared early during follow-up and are only partly attributable to weight loss with GLP-1 RA modulation. Concerning tirzepatide, the ongoing RCT SURMOUNT-MMO trial investigates its potential and the magnitude of CV benefit in adults with obesity and CVD.^89^ Retrospective data from a large US Medicare database (>85 000 obese individuals) suggested that semaglutide and tirzepatide may lead to a reduction in CVD outcomes, particularly heart failure (HF), atrial fibrillation (AF), arrhythmia, and peripheral vascular disease—findings that warrant confirmation in prospective studies.^90^ In this way, in a meta-analysis of 10 trials, including patients with AF undergoing catheter ablation, weight loss was associated with a greater reduction in recurrent AF after ablation. Newer pharmacological agents are under investigation and will add to the current body of evidence (*Table 2*). Evidence on sex-specific differences in the efficacy of anti-inflammatory CV therapies in patients with atherosclerosis is summarized in *Tables 1* and *2*.

**Table 2:** Newer pharmacological agents under investigation for the management of obesity

Pharmacological agent	Class	Administration route	Population	Sex-specific efficacy
*Phase III*				
Orforglipron	GLP-1 RA	Oral	Adults with obesity (BMI ≥ 30 kg/m^2^) or overweight (BMI ≥ 27 kg/m^2^) and ≥1 weight-related comorbidity without T2DM (ATTAIN-1) or with T2DM (ATTAIN-2) Adults with T2DM and obesity or overweight at increased CV risk (ACHIEVE-4)	No significant sex-based efficacy differences reported so far
CagriSema (cagrilintide + semaglutide)	Amylin analogue + GLP-1 RA	SC	Adults with obesity (BMI ≥ 30 kg/m^2^) or overweight (BMI ≥ 27 kg/m^2^) and ≥1 weight-related comorbidity without T2DM (REDEFINE 1) Adults with BMI ≥ 27 kg/m^2^ with T2DM (REDEFINE 2) and adults with established CVD (REDEFINE 3)	Not reported
Survodutide	Dual glucagon receptor agonist and GLP1-RA	SC	Adults with obesity (BMI ≥ 30 kg/m^2^) or overweight (BMI ≥ 27 kg/m^2^) and ≥1 weight-related comorbidity without T2DM (SYNCHRONIZE-1) or with T2DM (SYNCHRONIZE-2) or with established CVD (SYNCHRONIZE—CVOT)	Females showed larger reductions in bodyweight and waist circumference than males in a prespecified Phase 2 subgroup analysis of survodutide, but exact sex-specific numeric values are not reported^91^
Mazdutide	GIPR agonist and glucagon receptor agonist	SC	Adults with obesity (BMI ≥ 28 kg/m^2^) or overweight (BMI ≥ 24 kg/m^2^) and ≥1 weight-related comorbidity without T2DM (GLORY-1)	Not reported
Retatrutide	GIPR agonist, glucagon receptor agonist, and GLP1-RA	SC	Adults with obesity (BMI ≥ 30 kg/m^2^) or overweight (BMI ≥ 27 kg/m^2^) and ≥1 weight-related comorbidity without T2DM (TRIUMPH-1)	Not reported
*Phase II*				
Danuglipron	GLP1-RA	Oral	Adults with obesity (BMI ≥ 30 kg/m^2^) without T2DM	Not reported
Cagrilintide	Amylin receptor agonist	SC	Adults with obesity (BMI ≥ 30 kg/m^2^) or overweight (BMI ≥ 27 kg/m^2^) and hypertension or dyslipidaemia without T2DM	Not reported
PYY 1875	PYY RA	SC	Adults with BMI of 25–35 kg/m^2^ without T2DM	Not reported
Efinopegdutide	Dual glucagon receptor agonist and GLP-1 RA	SC	Adults with severe obesity (BMI ≥ 35 to ≤50 kg/m^2^) without T2DM	Not reported
Pemvidutide	Dual glucagon receptor agonist and GLP1 RA	SC	Adults with obesity (BMI ≥ 30 kg/m^2^) or overweight (BMI ≥ 27 kg/m^2^) and ≥1 weight-related comorbidity without T2DM	Not reported
Maridebart cafraglutide (AMG-133/MariTide)	GIPR antagonist and GLP1-RA	SC	Adults with obesity (BMI ≥ 30 kg/m^2^) or overweight (BMI ≥ 27 kg/m^2^) and ≥1 weight-related comorbidity with or without T2DM	Not reported
NNC0165-1875 + semaglutide	GLP1 RA + PYY RA	SC	Adults with BMI of 30–45 kg/m^2^ without T2DM	Not reported
Dapiglutide	GLP1 RA + GLP-2 RA	SC	Adults with BMI ≥ 30 kg/m^2^ without T2DM	Not reported
Semaglutide + bimagrumab	GLP1 RA + activin type II receptor blocking biologic	Semaglutide: SC Bimagrumab: IV	Adults with obesity (BMI ≥ 30 kg/m^2^) or overweight (BMI ≥ 27 kg/m^2^) and ≥1 weight-related comorbidity without T2DM	Not reported
S-309309	MGAT2	Oral	Adults with obesity (BMI ≥ 30 kg/m^2^) without T2DM	Not reported

BMI, body mass index; CV, cardiovascular; CVD: cardiovascular disease; GIPR, glucose-dependent insulinotropic peptide receptor; GLP-1 RA, glucagon-like peptide-1 receptor agonist; GLP-2, glucagon-like peptide-2 receptor agonist; IV, intravenous; MGAT2, monoacylglycerol transferase; PYY, peptide YY; SC, subcutaneous; T2DM, Type 2 diabetes mellitus.

### Surgical interventions

When lifestyle changes and pharmacological treatments fail to provide adequate results, bariatric surgery should be considered. According to current guidelines^92^ and recent review of global practices,^93^ the benefits of metabolic and bariatric surgery (MBS), especially when combined with multidisciplinary team (MDT) management, are quantified. However, there remains a need for additional RCTs, particularly in^68–70^ patients who are borderline candidates for MBS.^94^ Although MBS has been associated with reversal of cardiac remodelling—as soon as 12 months postsurgery and independent of BMI reduction^95^—long-term benefits appear limited by suboptimal control of BMI and BP at 5-year follow-up.^96^ Metabolic dysfunction-associated fatty liver disease (MAFLD) and metabolic dysfunction-associated steatohepatitis (MASH) are also highly prevalent in individuals with severe obesity. Unfortunately, no anthropometric measurements or laboratory tests have proven reliable in predicting the presence or severity of either condition. Once MAFLD is diagnosed, research from Canadian investigators has shown that women may be at higher risk of progressing to advanced stages of liver fibrosis.^97^

Metabolic and bariatric surgery is associated with significant decreases in the risk of Type 2 diabetes mellitus (T2DM) and CVD,^98^ MACE,^99,100^ new-onset HF, and MI,^101^ as well as all-cause mortality^101,102^—particularly in patients with T2DM.^103^ A recent 15-year follow-up study showed a substantial reduction in the progression from prediabetes to T2DM, particularly in women. The study included a cohort composed of 83% women and demonstrated that Roux-en-Y gastric bypass (RYGB) was more effective than sleeve gastrectomy (SG) in preventing diabetes progression.^104^ Importantly, women constituted at least 60% of the populations in most of the referenced MBS studies. Even among paediatric patients—43% of whom were female—MBS has been shown to significantly reduce morbidity in young adulthood.^105^

Two additional aspects of women’s CV health that have been positively influenced by MBS are reproductive and mental health. Nearly half of women with PCOS experience clinically significant anxiety. Although the prevalence of PCOS was low during the 4-year follow-up period after MBS, women with PCOS were more likely to report migraines, disinhibited eating behaviours, and anxiety, despite achieving comparable BMI outcomes to those without PCOS.^106^ Moreover, in a predominantly female cohort, a history of anxiety among patients undergoing MBS was associated with nearly a three-fold increase in the odds of developing hypertension^107^—a key contributor to adverse long-term CV outcomes.

## Conclusion and recommendations

Obesity and chronic inflammation play an important role and effect in the development of atherosclerosis in women. These effects are exaggerated by female-specific factors across different stages of life, yet current management strategies are still largely based on men-dominant evidence. Greater attention to body fat distribution, inflammatory burden, and women-specific risk factors is needed in everyday clinical practice. Improving research design and clinical care with a stronger focus on women is essential to achieve better CV outcomes.

## Recommendations:

1. **Screening and risk assessment**
  - Reproductive history documentation, including age at menarche/menopause, parity, pregnancy complications (preeclampsia, gestational diabetes, preterm delivery), PCOS, and infertility
  - Measurement of BMI and waist circumference or waist-to-hip ratio at baseline and annually
  - Measurement of cardiometabolic panels, including BP, fasting lipids, fasting glucose or glycosylated hemoglobin A1c, and smoking status
  - Screening for autoimmune and inflammatory conditions, such as CV risk enhancers
2. **Weight management intervention**
  - Setting individualized, measurable short-term goals that improve cardiometabolic markers and functions, such as dietary counselling and calorie awareness
  - Integration of social support and multidisciplinary teams
  - Reservation of pharmacotherapy or bariatric referral for women who do not achieve meaningful improvement with lifestyle measures
  - Coordination with obesity specialists and considering impacts on reproductive plans
  - Documentation of weight management goals and tracking progress at each visit
3. **Structured PA programmes**
  - Prescribe combined aerobic and resistance training tailored to baseline fitness and comorbidities.
  - Emphasize progressive increases with moderate-to-vigorous activity adapted to individual capacity.
  - Set concrete weekly activity goals and review barriers at follow-up visits.
**Women-specific considerations**
  - Account for life stage factors: pregnancy, postpartum recovery, menopausal symptoms, and musculoskeletal issues.
  - Address caregiving constraints and time barriers in programme design.
4. **Pregnancy-specific considerations**
**Prepregnancy screening**
  - Evaluate traditional risk factors plus prior pregnancy complications.
  - Optimize hypertension, lipids, diabetes, and weight before conception.
  - Refer to cardio-obstetrics for women with high baseline risk or prior adverse pregnancy outcomes.
**During pregnancy**
  - Emphasize healthy gestational weight gain tailored to prepregnancy BMI.
  - Prioritize nutrition, PA, and glycaemic control rather than weight loss.
  - Avoid weight-loss pharmacotherapies during pregnancy.
**Postpartum follow-up**
  - Arrange early cardiometabolic reassessment for women with hypertensive disorders, gestational diabetes, or substantial gestational weight gain.
  - Ensure secure handoff from obstetric to primary/cardiology care with documented CV risk reduction plan.
  - Continue surveillance into midlife with regular monitoring.
5. **Menopause management**
  - Perform CV risk assessment at menopause onset.
  - Document menopausal status and associated symptoms in CV risk profile.
6. **Inflammation management**
  - Recommend eating vegetables, whole grains, healthy fats, and lean proteins.
  - Manage autoimmune and systemic inflammatory disorders to reduce vascular risk.
  - Coordinate with rheumatology or specialty care for complex inflammatory conditions.
7. **Follow-up and monitoring**
  - Baseline and annual reassessment for women with risk factors
  - More frequent visits following acute events or medication changes
